# Prognostic Equations and Accuracy of a Total Score of Functional Independence Measure at Discharge for Different Diseases in a Convalescent Rehabilitation Ward

**DOI:** 10.7759/cureus.66509

**Published:** 2024-08-09

**Authors:** Shirou Mikayama, Takaaki Kubo, Tuyoshi Tahara, Masatoshi Nakamura, Fumika Oku, Kunihiko Kenmochi

**Affiliations:** 1 Department of Rehabilitation, Tobata Rehabilitation Hospital, Kitakyushu, JPN; 2 Faculty of Rehabilitation Sciences, Department of Physical Therapy, Nishikyushu University, Saga, JPN

**Keywords:** vertebral compression fractures, hip fracture, stroke, rehabilitation, functional independence measure

## Abstract

Objectives: Prognosis and goal setting from admission in the convalescent rehabilitation ward, supported by a multidisciplinary team, enhance rehabilitation and discharge support. Predicting functional independence measure (FIM) outcomes can further optimize these processes. This study aimed to develop prognostic equations for the motor FIM at discharge for stroke, hip fracture (HF), vertebral compression fractures (VCFs), and total knee arthroplasty (TKA), which are common diseases in patients admitted to convalescent rehabilitation wards, using multiple regression analysis, and to clarify the difference in the accuracy of the predicted motor FIM according to the disease.

Methods: This study included 965 patients admitted to our hospital. The objective variable consists of the motor FIM at discharge, and the explanatory variables were age, sex, days from onset to admission, total admission motor FIM, and total admission cognitive FIM. A stepwise multiple regression analysis was performed. The analysis of the difference in the accuracy of predicted motor FIM by disease used the absolute value of the residuals.

Results: The total motor FIM and cognitive FIM at admission were extracted for all four diseases included in this study. The absolute value of the residuals appeared to be more accurate for TKA, HF, stroke, and VCF in that order.

Conclusions: Although differences in the accuracy of the prediction equation were observed by disease, this prediction equation can be used as an approach to review the details of rehabilitation and discharge and can be tailored to each case.

## Introduction

The functional independence measure (FIM) is now used as the basis for the classification criteria for admission fees for the convalescent rehabilitation ward in the 2018 revision of reimbursement. It is one of the most reliable physical therapy assessments used among multiple professions in medical institutions [1].

The rehabilitation aims to improve the activities of daily living (ADL) of patients and help them return to their normal lives. Further, aiming for higher ADL capabilities within an appropriate number of days in the ward is now required, with the introduction of the performance index indicating that the number and the quality of rehabilitation services provided are being investigated [2].

Stroke, hip fracture (HF), vertebral compression fractures (VCFs), and total knee arthroplasty (TKA) are frequently the subject of rehabilitation, and efficiently improving ADL is crucial. Therefore, FIM should be used to evaluate the subject’s ADL abilities, which are predicted for the time of discharge or transfer. Additionally, appropriate rehabilitation diagnosis and prognosis of recovery should be made for the patient from admission to the convalescent rehabilitation ward, and goals should be set early on, considering rehabilitation support for the living environment [3]. A multidisciplinary medical team developed rehabilitation treatment to achieve this set goal. Therefore, more individualized rehabilitation and smooth discharge support are assumed to be provided if the prognosis of FIM can be predicted.

Multiple studies have been reported on predicting prognosis at discharge. Specifically, a study by Inouye [4] in patients with stroke revealed that multiple regression analysis using motor and cognitive FIM at admission may predict total FIM at discharge. Additionally, the addition of clinical measures, such as the trunk impairment scale [5], Stroke Impairment Assessment Set [6], and comorbidity index [7], to motor and cognitive FIM has been reported to improve the prediction accuracy of discharge FIM scores. A study by Mutai et al. [8] performed multiple regression analyses with discharge FIM as the objective variable and revealed that age, pre-morbid ability, stroke type, admission motor FIM, and cognitive FIM were predictors of discharge FIM. Moreover, Heruti et al. [9] reported that admission FIM can predict discharge FIM in HF.

Many of these studies focused on diseases to unify patient characteristics, and the only report, particularly on predictive FIM for orthopedic diseases, is the aforementioned study on patients with HF. We believe that predicting FIM at the time of discharge is necessary not only for cerebrovascular diseases but also for orthopedic diseases from the early stage of hospitalization to clarify intervention details and set goals such as returning home.

Therefore, this study aimed to develop prognostic equations for the motor FIM at discharge for stroke, HF, VCF, and TKA, which are common diseases in patients admitted to the convalescent rehabilitation ward, using multiple regression analysis, and to clarify the difference in the accuracy of the predicted motor FIM according to disease.

## Materials and methods

The study included 965 out of 1,165 patients with stroke, HF, VCF, and TKA admitted to our hospital from April 2019 to March 2022, excluding those admitted within 10 days.

Furthermore, stroke cases included patients with cerebral infarction (198 patients), cerebral hemorrhage (76 patients), and subarachnoid hemorrhage (18 patients). HF included patients who had suffered a femoral neck fracture, femoral condyle fracture, or bipolar hip arthroplasty (100 patients) and osteosynthesis (226 patients). VCF included patients who were corseted with conservative surgery after the injury. TKA included patients with osteoarthritis of the knee.

In all diseases, physical and occupational therapy interventions were provided as rehabilitation. Physical therapy included strength training, balance training, standing, transferring, and walking, while occupational therapy included ADL training such as standing, transferring, toileting, changing, and bathing.

The study was conducted retrospectively from medical records to investigate the patient's age, sex, number of days from onset to admission, number of days from admission to discharge, the average daily rehabilitation time (minutes), gross motor FIM on admission (13-91 points), gross cognitive FIM on admission (5-35 points), and gross motor FIM on discharge (13-91 points), which can be assessed in common for the four diseases under study. The admission FIM, which was studied retrospectively, was assessed jointly by physical therapists, occupational therapists, and nurses within three days of admission. The discharge FIM was a reassessment of the subject patient's abilities as of the date of discharge by the staff who assessed the patient at the time of admission.

Univariate analysis of basic information and total admission motor FIM, admission total cognitive FIM, and total discharge motor FIM were performed by disease for statistical analysis. The objective variable includes the motor FIM at discharge, and the explanatory variables were age, sex, days from onset to admission, total admission motor FIM, and total admission cognitive FIM, referring to a previous study by Tokunaga et al. [10]. Stepwise multiple regression analysis (variable increasing and decreasing method) was performed. Sex (male/female) was converted to a dummy variable for analysis in the nominal measures in this study.

Total motor FIM at discharge was used as the objective variable as used in the performance index {(motor FIM at discharge - motor FIM at admission)/(maximum days in hospital/number of days in hospital)}, which is the institutional standard for convalescent rehabilitation wards.

The analysis of the difference in the accuracy of predicted motor FIM by disease (stroke, HF, VCF, and TKA) used the coefficient of determination, root mean square deviation (RMSD), and the absolute value of the residuals. RMSD represents the root mean square deviation of the difference between the predicted and measured values and is considered one measure of predictive power [11]. Previous studies conducted comparisons using residuals, squares of residuals, and absolute values of residuals [12-15]. This study used the absolute value of the residuals because this study aimed to compare prediction formulas and because the actual scores are more important than the systematic error. One-way repeated measures analysis of variance (ANOVA) and the Tukey-Kramer test as a post hoc test were also used to compare absolute values of the residuals by disease.

SPSS (IBM Corp., Armonk, NY) was used for each statistical treatment, and the significance level was set at 5%. Variance inflation factor (VIF) was calculated, and multicollinearity was considered to be present when VIF was >10.

Ethical approval

The study was conducted by the tenets of the Declaration of Helsinki and was approved by the Ethics Committee of the Tobata Rehabilitation Hospital (approval number: 103).

## Results

Basic attributes of participants

Table 1 shows the age, sex, days from onset to admission, mean total admission motor FIM, total admission cognitive FIM, total discharge motor FIM, number of days spent in hospital, and hours of rehabilitation per day of the participants.

**Table 1:** Basic attributes by disease. Data are presented as mean (SD). HF: hip fracture; VCF: vertebral compression fracture; TKA: total knee arthroplasty; FIM: functional independence measure.

Parameter	Stroke	HF	VCF	TKA
	(n=292)	(n=326)	(n=256)	(n=91)
Age	76.8±11.6	83.3±10.4	83.3±9.5	73.8±9.3
Sex (male/female)	157/135	71/255	63/285	22/69
Days from onset to hospitalization	26.6±15.4	20.7±10.4	13.6±11.3	16.8±4.3
Total admission motor FIM	41.6±23.7	44.1±17.5	41.9±17.8	71.0±8.5
Total admission cognitive FIM	20.3±10.0	25.2±8.4	28.3±6.7	33.9±8.5
Total discharge motor FIM	58.9±26.3	65.8±18.5	70.3±17.0	84.8±5.6
Number of days spent in hospital	70.2±35.5	56.4±22.7	53.5±21.4	40.6±17.0
Hours of rehabilitation per day (minutes)	157.4±26.4	114.1±22.7	116.1±21.4	133.3±9.4

Factors associated with motor FIM at discharge

The results of multiple regression analysis, with the objective variables as total discharge motor FIM, age, total admission motor FIM, and total admission cognitive FIM were extracted for stroke; total admission motor FIM and total admission cognitive FIM for hip fracture; sex, days from onset to admission, total admission motor FIM, and total admission cognitive FIM for TKA; sex, total admission motor FIM, and total admission cognitive FIM for VCF (Tables 2-5).

**Table 2:** Multiple regression analysis by stroke. Stepwise multiple regression analysis (variable increasing/decreasing method) was performed with the objective variable being total discharge motor FIM and the explanatory variables being age, sex, number of days from onset to hospitalization, total admission motor FIM, and total admission cognitive FIM. B: Partial regression coefficient; CI: confidence interval; VIF: variance inflation factor; FIM: functional independence measure.

Stroke	B	95% CI for B		P-value	VIF
Variables		Lower bound	Upper bound		
Intercept	24.957	14.169	35.746	<0.01	-
Age	-0.143	-0.267	-0.019	<0.05	1.086
Total admission motor FIM	0.757	0.666	0.848	<0.01	2.464
Total admission cognitive FIM	0.642	0.426	0.857	<0.01	2.428

**Table 3:** Multiple regression analysis by HF. Stepwise multiple regression analysis (variable increasing/decreasing method) was performed with the objective variable being total discharge motor FIM and the explanatory variables being age, sex, number of days from onset to hospitalization, total admission motor FIM, and total admission cognitive FIM. B: partial regression coefficient; CI: confidence interval; VIF: variance inflation factor; HF: hip fracture; FIM: functional independence measure.

HF	B	95% CI for B		P-value	VIF
Variables		Upper bound	Lower bound			
Intercept	25.002	20.770	29.235	<0.01	-
Days from onset to hospitalization	-0.126	-0.233	-0.019	<0.05	1.004
Total admission motor FIM	0.526	0.432	0.620	<0.01	2.233
Total admission cognitive FIM	0.817	0.620	1.014	<0.01	2.236

**Table 4:** Multiple regression analysis by VCF. Stepwise multiple regression analysis (variable increasing/decreasing method) was performed with the objective variable being total discharge motor FIM and the explanatory variables being age, sex, number of days from onset to hospitalization, total admission motor FIM, and total admission cognitive FIM. B: partial regression coefficient; CI: confidence interval; VIF: variance inflation factor; VCF: vertebral compression fracture; FIM: functional independence measure.

VCF	B	95% CI for B		P-value	VIF
Variables		Lower bound	Upper bound		
Intercept	23.859	16.851	30.866	<0.01	-
Sex	3.883	0.399	7.367	<0.05	1.024
Days from onset to hospitalization	-0.188	-0.325	-0.051	<0.01	1.087
Total admission motor FIM	0.388	0.271	0.469	<0.01	1.413
Total admission cognitive FIM	1.083	0.829	1.337	<0.01	1.341

**Table 5:** Multiple regression analysis by TKA. B: partial regression coefficient; CI: confidence interval; VIF: variance inflation factor; TKA: total knee arthroplasty; FIM: functional independence measure. Stepwise multiple regression analysis (variable increasing/decreasing method) was performed with the objective variable being total discharge motor FIM and the explanatory variables being age, sex, number of days from onset to hospitalization, total admission motor FIM, and total admission cognitive FIM.

TKA	B	95% CI for B		P value	VIF
Variables		Lower bound	Upper bound		
Intercept	41.368	32.805	49.932	<0.01	ー
Sex	-2.023	-3.852	-0.193	<0.05	1.015
Total admission motor FIM	0.172	0.074	0.271	<0.01	1.485
Total admission cognitive FIM	0.962	0.666	1.258	<0.01	1.469

No multicollinearity was observed.

The prediction equation for stroke was as follows: 24.975 - age × 0.143 + 0.757 × total admission motor FIM + 0.642 × total admission cognitive FIM.

The predictive equation for HF was as follows: 25.002 - 0.126 × days from onset to admission + 0.526 × total admission motor FIM + 0.817 × total admission cognitive FIM.

The predictive equation for VCF was as follows: 23.859 + sex (male = 1 and female = 2) × 3.883 - 0.188 × days from onset to admission + 0.388 × total admission motor FIM + 1.083 × total admission cognitive FIM.

The prediction equation for TKA was as follows: 41.368 - sex (male = 1 and female = 2) × 2.023 + 0.172 × total admission motor FIM + 0.962 × total admission cognitive FIM.

Accuracy of prediction equations by disease

The values for stroke, hip fracture, VCF, and TKA were 0.799, 0.689, 0.499, and 0.520, respectively, in terms of the coefficient of determination.

**Figure 1:**
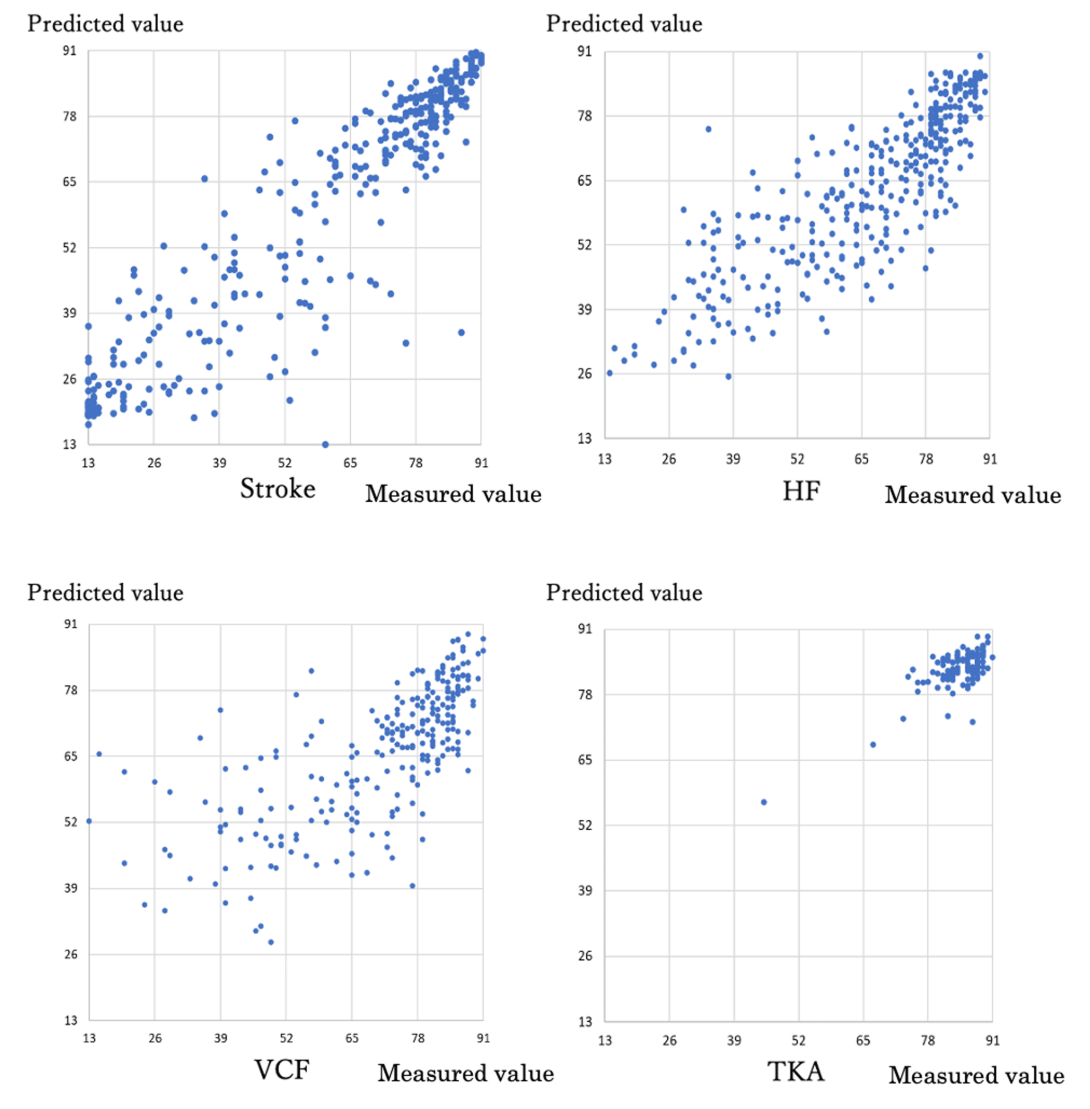
Scatterplot of measured and predicted motor FIM totals at discharge for each disease-specific prediction equation. HF: hip fracture; VCF: vertebral compression fracture; TKA: total knee arthroplasty; FIM: functional independence measure.

**Figure 2:**
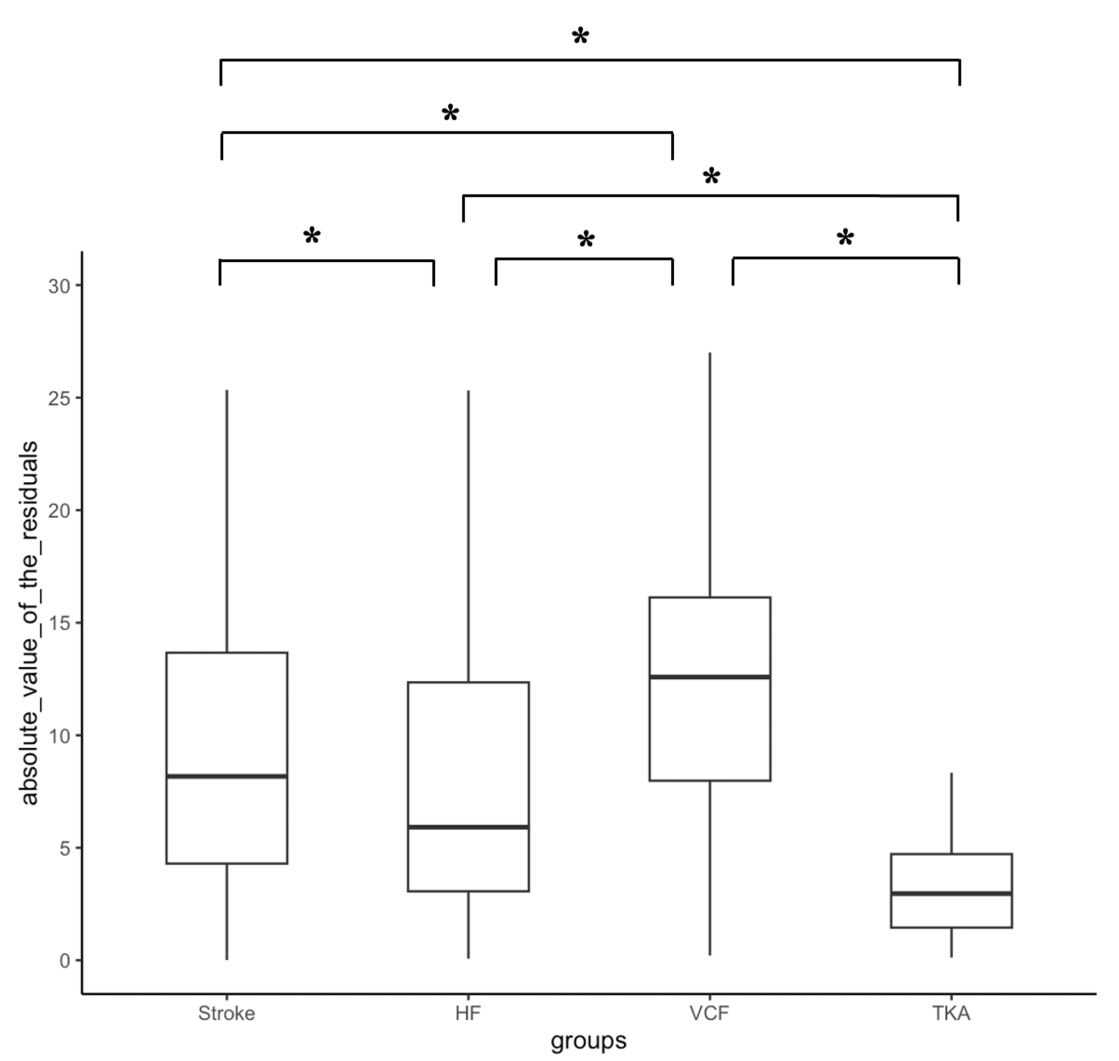
Absolute value of residuals. *: Tukey-Kramer test, P < 0.05. The box-and-whisker diagram shows the median (middle value), interquartile range (IQR, the middle 50% of the data), and maximum and minimum values. Asterisks indicate significant differences in comparing absolute values of the residuals by disease. HF: hip fracture; VCF: vertebral compression fracture; TKA: total knee arthroplasty.

We calculated the predicted values of total discharge motor FIM, scatter plots of the measured and predicted values, the absolute difference between the measured and predicted values (residuals), and the RMSD of the residuals from the prediction equation for discharge motor FIM using the regression coefficient estimates of multiple regression analysis (Table 6 and Figures 1, 2).

**Table 6:** Accuracy of prediction equations by disease. HF: hip fracture; VCF: vertebral compression fracture; TKA: total knee arthroplasty; R^2^: coefficient of determination; RMSD: root mean square deviation.

Accuracy	Stroke	HF	VCF	TKA
	(n=292)	(n=326)	(n=256)	(n=91)
R^2^	0.799	0.689	0.499	0.520
Absolute value of residual	9.7±7.2	8.2±6.8	13.1±7.6	3.4±2.6
RMSD	11.952	10.147	15.226	4.289

Both the absolute value of the residuals and the RMSD appeared to be more accurate for TKA, HF, stroke, and VCF in that order.

## Discussion

This study determined the factors associated with discharge motor FIM for stroke, HF, VCF, and TKA, which are common diseases in patients admitted to a convalescent rehabilitation ward, developed prognostic equations, and discussed differences in the accuracy of predicted motor FIM by disease. The findings of this study emphasize the importance of considering a different set of predictors when assessing functional independence in patients with stroke, HF, TKA, and VCF. Motor and cognitive FIM scores at admission consistently emerge as significant predictors in various conditions and are useful in predicting functional outcomes. Also, the absence of a VIF suggests that the selected predictor variables provide unique and complementary information, enhancing the robustness of the regression models.

Accuracy of predicted motor FIM by disease

Multiple correlation coefficients are frequently used in multiple regression analysis to compare accuracy. The prediction equations developed in this study for FIM at discharge for stroke and HF are considered to be within the accuracy of existing prediction equations based on the FIM prediction at discharge by Fujiwara et al. [5] (R^2^ = 0.66-0.75), Tsuji et al. [6] (R^2^ = 0.68), and Sonoda et al. [16] (R^2^ = 0.7-0.9). Wada et al. [17] also compared the accuracy of the prediction equations using the absolute value of the residuals obtained by subtracting the predicted value from the measured value of the total motor FIM at discharge. The results of the accuracy comparisons conducted in this study showed that TKA, HF, stroke, and VCF were more accurate in that order.

Stroke requires consideration of severity, trunk impairment scale, Stroke Impairment Assessment Set, and comorbidity index in terms of prognosis. Age and cognitive function are associated with motor function prognosis, with higher cognitive function and lower age associated with better prognosis [16,17].

Preoperative factors associated with length of stay and outcomes for HF have influenced the length of stay from admission to surgery, surgical technique, complications, sex, physical function, mental function, nutritional status, and pre-fracture residential status [18,19].

Previous studies on the functional prognosis of VCF have revealed that age, posterior wall injury, posterior curvature angle, crush rate on radiographic images, number of VCF, and time of treatment initiation are associated [20]. The predicted value may have been underestimated in the case of VCF, where the accuracy was low because the corset was incomplete when the patient was admitted. The patient had the physical function to leave the bed but was unable to do so. Further, the corset may not have been removed by the time of discharge, and the scores for the bathing and changing motor FIM items did not increase, causing lower measured values than predicted. Therefore, we believe that the results for VCF were less accurate.

The TKA tended to have higher total motor FIM on admission than other diseases. ADL and mobility in many cases were gained since admission to the convalescent rehabilitation ward. The accuracy of the prediction was considered high because the total discharge motor FIM was close to that for the full score of 91.

Scattered factors are associated with the prognosis of stroke, HF, and VCF, as mentioned above, and most of these previous studies have attempted to examine functional prognosis using multiple factors. However, the Japanese guidelines for the management of stroke (2015) state that “simply increasing the number of variables for prediction does not necessarily improve prediction accuracy,” and the benefits of using as simple a prediction method as possible have been shown [21,22]. This study only used a minimal number of basic clinical indicators because the focus was on predicting the prognosis of motor FIM at discharge from an early stage. Therefore, we believe that the index can be used easily from an early stage, enabling the selection of rehabilitation programs according to the predictions and early determination of patients to be excluded from the performance index in the convalescent rehabilitation ward. In addition, a study on prognostic prediction of FIM at discharge using machine learning has been reported [23]. Machine learning improves the accuracy of prognosis prediction. Thus, applying it clinically is difficult because it is sometimes a black box, it is unclear what logic is used in statistical processing, and the contribution of explanatory variables is unknown. Compared to machine learning, multiple regression analysis has slightly lower prediction accuracy. It remains a well-established method because the weighting of factors can be shown as standardized partial regression coefficient values, and the results can be obtained immediately using statistical software.

Limitations of this study include the small number of explanatory variables entered and the type of surgery, illness severity, or progress during hospitalization, which was not considered. However, taking this prediction formula as an average value, using it as an approach to reexamine the content of rehabilitation and discharge, and making predictions according to each case is crucial because a group trend can be predicted.

## Conclusions

This study developed prognostic equations for discharge motor FIM scores across four common conditions in the convalescent rehabilitation ward: stroke, HF, VCF, and TKA. The findings highlight that different predictor variables are important for each condition, though admission motor and cognitive FIM scores were consistently significant predictors. The accuracy of the prediction models was comparable to existing equations in the literature, with accuracy varying by disease. While more complex models exist, this study aimed for simplicity to enable early prognosis prediction and rehabilitation planning. These equations offer a useful tool for early rehabilitation planning and outcomes assessment in convalescent care while acknowledging the need for individualized consideration in each case.
